# A proteomic approach for the identification of biomarkers in endometrial cancer uterine aspirate

**DOI:** 10.18632/oncotarget.22725

**Published:** 2017-11-30

**Authors:** Blendi Ura, Lorenzo Monasta, Giorgio Arrigoni, Cinzia Franchin, Oriano Radillo, Isabel Peterlunger, Giuseppe Ricci, Federica Scrimin

**Affiliations:** ^1^ Institute for Maternal and Child Health-IRCCS “Burlo Garofolo”, Trieste, Italy; ^2^ Department of Biomedical Sciences, University of Padova, Padova, Italy; ^3^ Proteomics Center, University of Padova and Azienda Ospedaliera di Padova, Padova, Italy; ^4^ Department of Medical, Surgery and Health Sciences, University of Trieste, Trieste, Italy

**Keywords:** endometrial neoplasms, uterine aspirate, two dimensional electrophoresis, mass spectrometry, biomarkers

## Abstract

Endometrial cancer arises from the endometrium. It has a slow progression and a reported survival rate of 75%. The identification of soluble biomarkers in the uterine aspirate may be very useful for its early diagnosis. Uterine aspirates from 10 patients with endometrial cancer and 6 non-endometrial cancer controls were analyzed by two-dimensional gel electrophoresis coupled with mass spectrometry and western blotting for data verification. A total of 25 proteins with fold change in %V ≥2 or ≤0.5 in intensity were observed to change significantly (P<0.05). From the discovery phase, four proteins (costars family protein ABRACL, phosphoglycerate mutase 2, fibrinogen beta chain, annexin A3) were found to be present in the uterine aspirate of endometrial cancers and not in healthy aspirates. Western blotting verification data demonstrated that costars family protein ABRACL, phosphoglycerate mutase 2 were present only in endometrial cancer uterine aspirate while fibrinogen beta chain, annexin A3 were also present in healthy aspirates. To our knowledge, phosphoglycerate mutase 2 has not been previously associated with endometrial cancer. In this study we demonstrate that uterine aspirates are a promising biological fluid in which to identify endometrial cancer biomarkers. In our opinion proteins like costars family protein ABRACL and phosphoglycerate mutase 2 have a great potential to reach the clinical phase after a validation phase.

## INTRODUCTION

Endometrial cancer (EC) is the most common gynecological malignant neoplasm [1, 2].

In Italy it represents the fourth malignant tumor in women (5% of all tumors) after breast, colon and lung cancers, and the third if we consider women between 50 and 69 years of age (7%). Its incidence shows a slight upward time trend (+ 0.7% / year), while mortality has decreased in the last 10 years with the 5-year survival rate rised from 73% in 1990 to 77.5% in 2007 [3]. Nowadays, the majority of cases are diagnosed at early stages, while only 30% of EC patients are diagnosed at an advanced stage of the disease [4].

Abnormal uterine bleeding (AUB) is generally the first sign of the disease but AUB occurs in almost all women over the age of 45. Other symptoms include pain, bleeding, infection, and uterine perforation. Diagnosis relies on the results of the endometrial biopsy.

Early detection improves the chances that the endometrial cancer will be treated successfully.

Several risk factors are already known and include: diabetes mellitus, breast cancer, dietary, obesity, high levels of estrogen, and increasing age [5, 6].

Studies have established the involvement of defects in DNA mismatch repair genes, microsatellite instability, and mutations in the PTEN and K-ras and/or B-catenin genes and p53 suppressor gene with mutation of Her-2/neu in endometrial cancer physiopathology [7].

Proteins play a key role in several cellular processes and are often associated with diseases, including cancer. The search for EC biomarkers has been mostly based on tissue analyses. Li and colleagues identified CypA, E-FABP, CAPS as potential EC-associated proteins by using 2-DE coupled with mass spectrometry [8]. Multiple members of peroxiredoxin [9], members of the annexin family [9], prohibitin 2 and bone marrow stromal antigen 2 [10] were identified by proteomics technique as potential biomarkers or potential therapeutic targets in endometrial cancer. However, the detection of these proteins has not led to their use as biomarkers in clinical practice.

Biological fluids are rich with proteins and the different abundance of these proteins can indicate the presence of tumors and are thus valuable markers for the development of noninvasive diagnostic tests [11]. A little number of proteomic studies have been performed on uterine aspirates without a focus on EC biomarkers [12, 13].

Martinez-Garcia *et al.* conducted the first study focused on EC uterine aspirate using LC-PRM for biomarker identification [14].

An ideal biomarker research should be based on three sequential steps: discovery, verification and validation [15]. Obviously, the discovery phase produces a long list of biomarkers using a limited number of samples, that will need to be verified before entering the validation phase.

Two-dimensional gel electrophoresis (2-DE) is a powerful method for quantitative comparative proteomic studies, able for simultaneous resolution of thousands of proteins including isoform or protein PTM. 2-DE was used for the identification of biomarkers for several types of cancers [16, 17] but also to give more detailed information about these proteins as the presence of alternative splicing and/or isoforms.

The aim of the present study is the identification and verification of soluble biomarkers in the uterine aspirates of ECs, which could be then verified in a further validation phase.

## RESULTS

### 2-DE and MS

**Figure 1:**
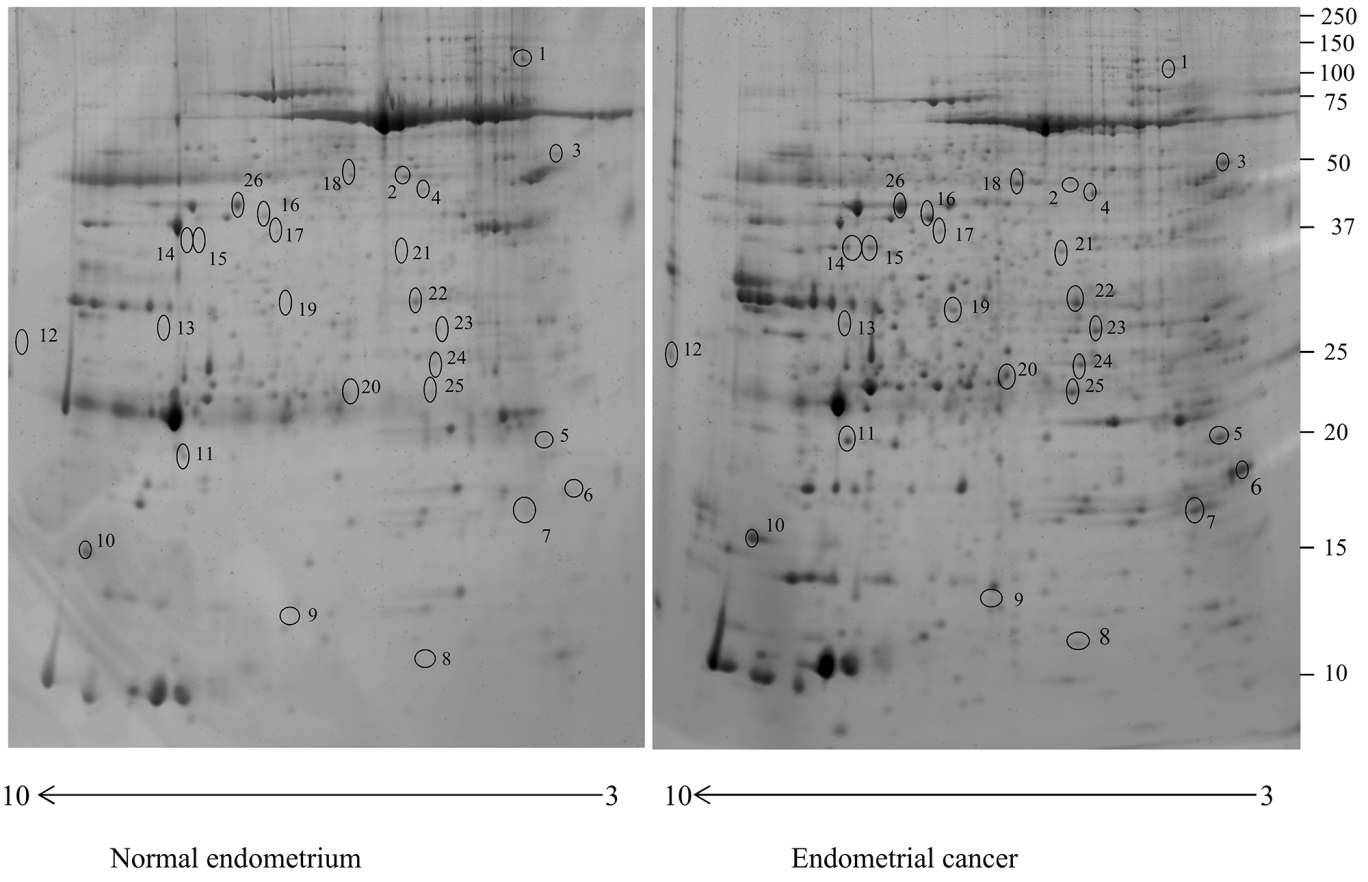
Two-dimensional electrophoresis map of the normal endometrium uterine aspirate and endometrial cancer uterine aspirate proteome Immobilized pH gradient pH 3-10 non-linear strips were used for the first dimension and 12% polyacrylamide gels were used for the second dimension.

In the present study, the uterine aspirate of six non-EC controls and ten women suffering from EC were resolved using 2-DE. More than a thousand protein spots were detected in each gel. For 2-DE analysis, 40 gels were used and the effectiveness of average-matching between each gel and the corresponding “Master gel” was 80% of the total number of spots. The analysis revealed 28 protein spots with different abundance in the uterine aspirate of the ECs, which corresponded to 25 proteins. Only protein spots significantly different between EC and control (P<0.05) and with a fold change in %V ≥2 or ≤0.5 were considered (Figure 1).

These proteins were identified by MALDI-TOF/TOF and LTQ-Orbitrap XL, and database searched against the human section of the UniProt database (version 20150401; 90,411 sequences) (Table 1).

**Table 1:** List of proteins with significantly different abundance in the uterine aspirate of the endometrial cancer compared to the aspirate of healthy controls, as identified by MALDI-TOF/TOF or LTQ-Orbitrap XL mass spectrometry, and classified by their corresponding molecular function. Only the most relevant molecular and cellular functions are reported

Accession no	Spot no	Protein description	Gene symbol	Protein score	Molecular function	Fold change^a^	P-value
Q9P1F3	8	Costars family protein ABRACL	ABRACL	52.25	Not known	490	0.0273
P02675	21	Fibrinogen beta chain	FGB	246.69	Binding	400	0.0050
P15259	20	Phosphoglycerate mutase 2	PGAM2	130.55	Catalytic activity	230	0.0431
P06733	15	Alpha-enolase	ENO1	339.27	Catalytic activity	220	0.0431
P12429	23	Annexin A3	ANXA3	480.59	Binding	180	0.0273
P20073-2	13	isoform 2 of Annexin A7	ANXA7	366.98	Binding	150	0.0412
P06733	14	Alpha-enolase	ENO1	50.65	Catalytic activity	120	0.0431
A6NIW5	7	Peroxiredoxin 2, isoform CRA_a	PRDX2	138.93	Antioxidant activity	60	0.0431
A0A087WT99	19	Ester hydrolase C11orf54	C11orf54	76.43	Catalytic activity	60	0.0431
P04406	12	Glyceraldehyde-3-phosphate dehydrogenase	GAPDH	291.10	Catalytic activity	50	0.0422
P28072	5	Proteasome subunit beta type-6	PSMB6	82.33	Catalytic activity/binding	20	0.0251
P48637	4	Glutathione synthetase	GSS	506.38	Catalytic activity	15	0.0277
J3KPD9	10	Nucleoside diphosphate kinase B	NME2	330.20	Catalytic activity	15	0.0277
Q7L266-2	9	Isoform 2 Isoaspartyl peptidase/L-asparaginase	ASRGL1	126.66	Catalytic activity	11.5	0.0431
P00558-2	25	Isoform 2 of Phosphoglycerate kinase 1	PGK1	132.98	Catalytic activity	10	0.0180
P31146	18	Coronin-1A	CORO1A	70.29	Binding	8.5	0.0431
P16930-2	17	isoform 2 of Fumarylacetoacetase	FAH	50.32	Catalytic activity	7	0.0277
F5H3C5	11	Superoxide dismutase	SOD2	108.73	Catalytic activity	5	0.0218
Q13938-3	6	isoform 2 of Calcyphosin	CAPS	235.58	Binding	5	0.0431
Q06323-3	24	isoform-3 Proteasome activator complex subunit 1	PSME1	73.07	Catalytic activity	3.3	0.0180
P30101	3	Protein disulfide-isomerase A3	PDIA3	253.77	Catalytic activity	3.3	0.0277
O75874	16	Isocitrate dehydrogenase [NADP] cytoplasmic	IDH1	698.72	Catalytic activity	3.2	0.0180
P06733	26	Alpha-enolase	ENO1	373.86	Catalytic activity	2.9	0.0284
P07195	22	L-lactate dehydrogenase B chain	LDHB	239.73	Catalytic activity	2	0.0241
Q96KP4	2	Cytosolic non-specific dipeptidase	CNDP2	186.34	Catalytic activity	0.3	0.0116
A0A0C4DGB6	1	Serum albumin	ALB	183.91	Binding	0.1	0.0249

^a^Fold change was defined as the ratio of the mean %V according to the formula %V=Vsingle spot/Vtotal spot of cases vs. controls.

We performed a ROC analysis to determine the sensitivity and specificity of each biomarker. The best-performing individual protein controls (sensitivity 100%, specificity 100%) (Supplementary Table 2) were: PDIA3, isoform 2 of CAPS, isoform CRA_a PRDX2, ABRACL, NME2, Isoform 2 ASRGL1, GAPDH, isoform 2 ANXA7, ENO1, CORO1A, C11orf54, PGAM2, FGB, ANXA3, PGK1. Of these, ABRACL, PGAM2, FGB, ANXA3 were present only in EC. Based on this data we decided to validate ABRACL, PGAM2, FGB, ANXA3 by western blotting.

### Functional analysis of the EC aspirate proteome

**Figure 2:**
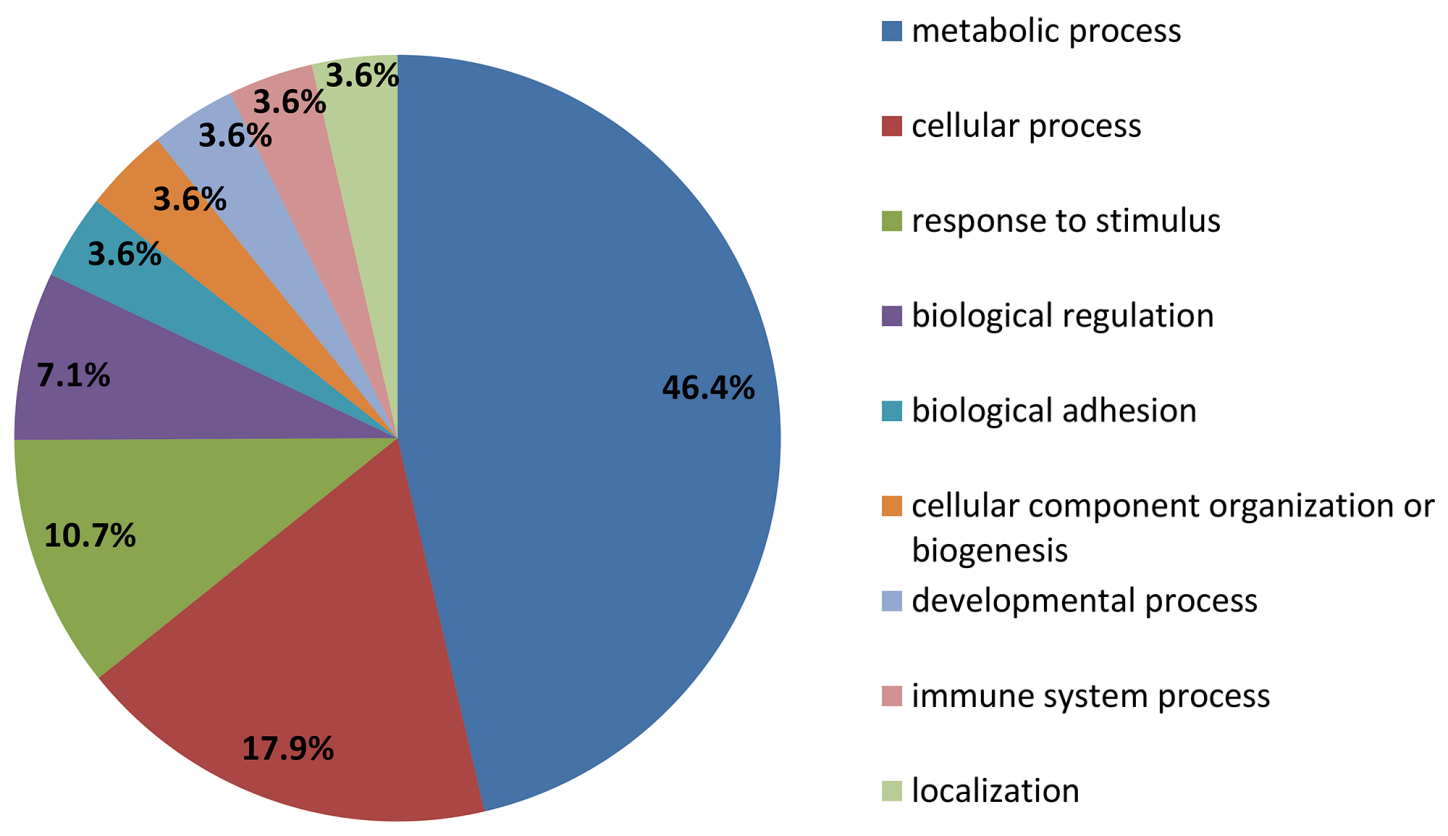
PANTHER classification of proteins upregulated in leiomyoma according to their biological process and molecular function

**Figure 3:**
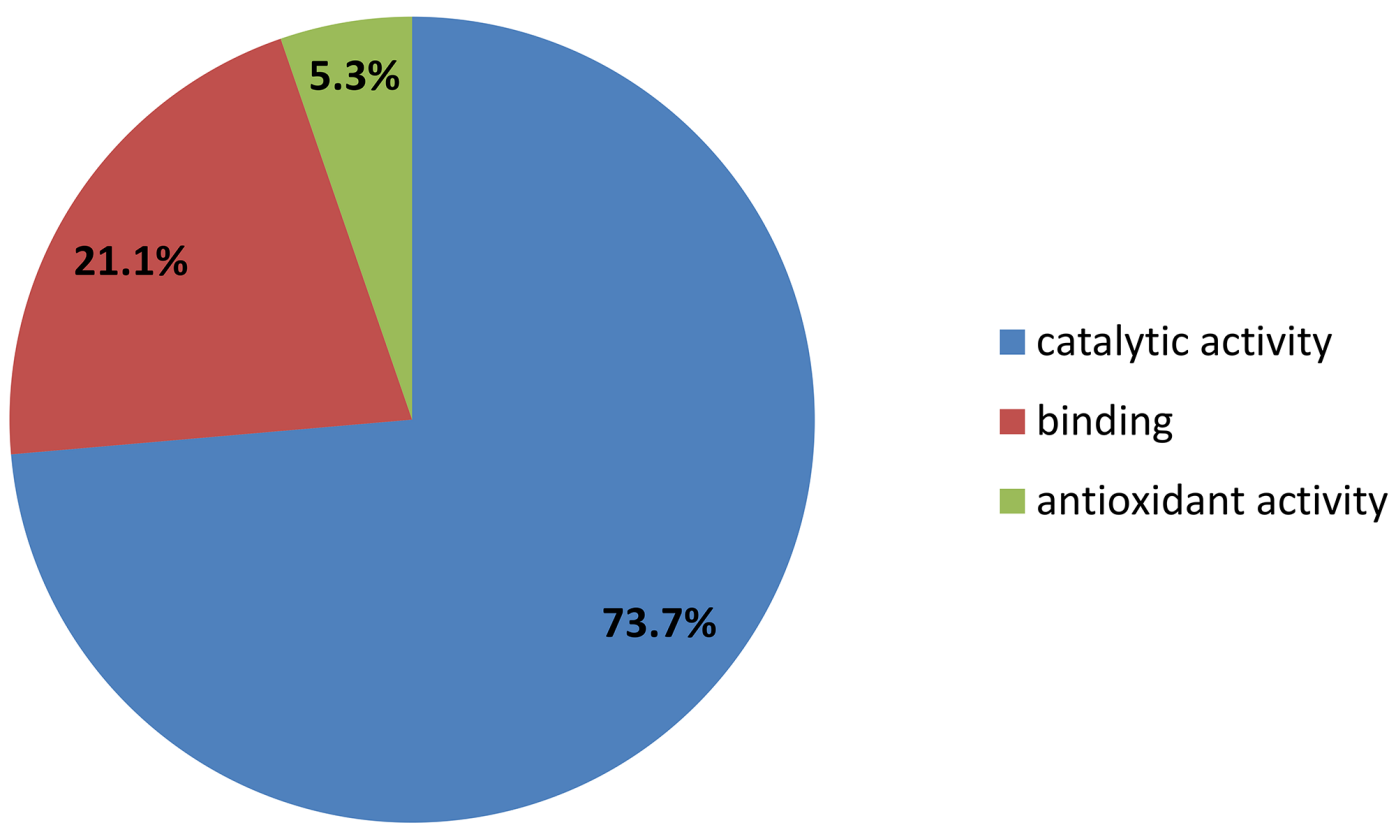
PANTHER classification of proteins upregulated in leiomyoma according to their molecular function

The 25 identified proteins were divided into groups, based on the PANTHER classification system, according to their biological processes and molecular functions. In terms of biological processes, proteins were grouped into four main categories: metabolic process, cellular process, response to stimulus and biological regulation (Figure 2). For molecular function the proteins were grouped into three categories: catalytic activity, binding, antioxidant activity (Figure 3). Fifteen of these proteins (PGAM2, IDH1, C11orf54, GSS, NME2, GAPDH, PSME1, PDIA3, FAH, PGK1, LDHB, ASRGL1, SOD2, ENO1, CNDP2) were found to be associated with the catalytic activity, according to the PANTHER prediction (Table 1). These data show the involvement of different types of enzymes in endometrial cancer disease.

### Western blotting analysis

**Figure 4:**
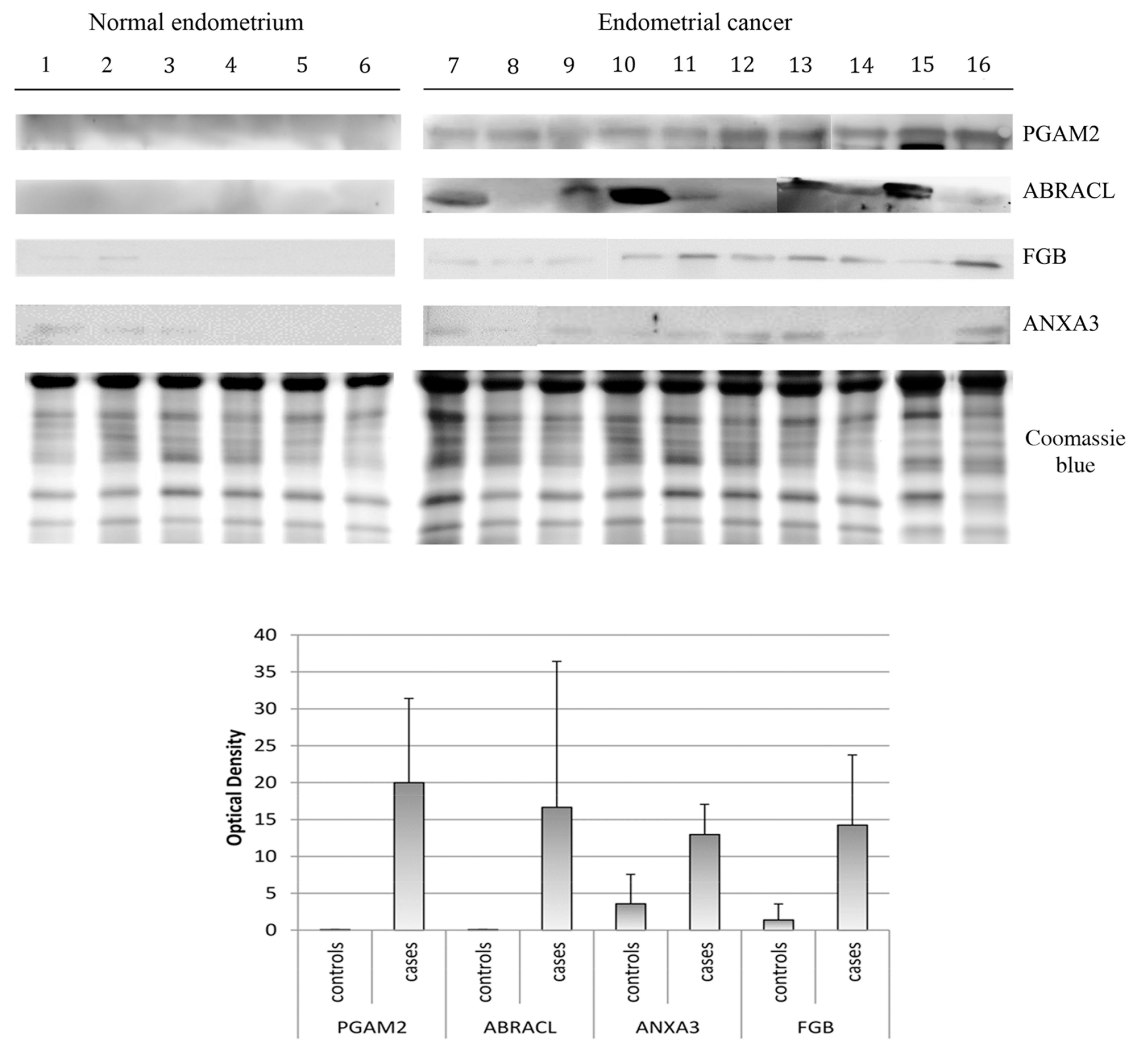
Western blot analysis of PGAM2, ABRACL, ANXA3, FBG normal endometrium uterine aspirate and endometrial cancer uterine aspirate The intensities of the immunostained bands were normalized with the protein intensities measured by Coomassie blue from the same blot. The bar graph shows the relative expression (band density) of proteins in the normal endometrium uterine aspirate and endometrial cancer uterine aspirate. The results are shown as a histogram (mean) with error bars representing the standard deviation. All differences were observed to be significant (Wilcoxon signed-rank test for matched samples, P<0.05).

In this study, western blot (WB) analysis from ten endometrial cancer vs six normal endometrium was used to validate the abundance of the four proteins which discriminate unambiguously between cases and controls (ABRACL, PGAM2, FGB, ANXA3). As seen from Figure 4, only ABRACL and PGAM2 are able to unambiguously discriminate the controls, from the EC patients.

The western blot data verified the presence of PGAM2 in all EC patients, behaving as a marker for G1, G2, G3, G2-G3 and serous papillary adenocarcinoma EC phase.

For ABRACL, western blotting verified its presence in eight EC patients G1, G2, G3, serous papillary adenocarcinoma phase, while we did not observe the presence of ABRACL in one G2 and one G2-G3 phase.

In case of FGB, we found the protein in all the EC samples but this protein was also present in the samples of two healthy subjects (hyperplastic endometrium and normal endometrium). For ANXA3 we found this protein in nine EC samples. This protein was absent in G3 samples and was found in three healthy patients (hyperplastic endometrium and two normal endometrium). FGB and ANXA3 were not able to discriminate the normal endometrium from the EC, although their expression level is significantly increased in EC.

## DISCUSSION

To date, several studies have been carried out for the identification of EC biomarkers, but no protein has yet reached the stage of clinical application. This may be related to the lack of studies on biofluids for the identification of soluble biomarkers and overall to the difficulty in performing such kind of studies on large cohorts of samples for the validation phase. The studies conducted until now focused on tissues and on plasma/serum [18, 19]. However, the research for biomarkers in serum/plasma is very difficult due to the large dynamic range of protein concentration and the low concentration of the potential biomarkers [20, 21].

To our knowledge, our study is the first using 2-DE with SYPRO Ruby and data verification by western blotting in the EC uterine aspirate. We identified 25 potential biomarkers, but only four of them were present only in ECs. Further data validation by western blotting showed that ABRACL and PGAM2 fully discriminate the healthy from EC samples. Our verification data showed the presence of PGMA2 in all EC patients. This protein has the potential to be identified as a biomarker for all EC phases and in serous papillary adenocarcinoma. While ABRACL verification data showed the presence of this protein in eight EC patients, only two samples (G2 and G2-G3 phase) did not show the presence of this protein, and this may be associated with the low abundance of these protein in the samples. However, the protein may be a potential biomarker for EC in G1, G2, G3 and serous papillary adenocarcinoma.

Maxwell GL at colleagues conducted a detailed study on the proteomic profile of EC and endometrium samples using label-free GeLC-MS/MS [9]. We compared our data with their data. The comparison confirmed that the expression of FGB, ENO1, ANXA3, PRDX2, GAPDH, PSMB6, GSS, ASRGL1, PGK1, CORO1A, PSME1, PDIA3, IDH1, LDHB was upregulated. In Martinez-Garcia *et al.*, ENO1 was confirmed as an up-regulated protein in endometrial cancer [14].

DeSouza L *et al.* studied the proteomic profile of EC and endometrium samples using labeled tags iTRAQ and cICAT: NME2, SOD2, CAPS were upregulated while ALB was down-regulated [22].

Our proteomic study revealed the dysregulation of fifteen metabolic enzymes showing a clear disruption of cancer metabolism.

ABRACL is a 82 amino acid protein that increase actin dynamics and cell motility [23].

PGAM2 is a glycolytic enzyme that catalyzes the reversible conversion of 3-phosphoglycerate (3-PG) to 2-phosphoglycerate, which is highly expressed in muscle tissues [24]. This enzyme increase NADPH homeostasis in response to the oxidative stress that impacts cell proliferation and tumor growth [25]. Further studies with larger groups of patients and controls are needed for the validation of ABRACL and PGAM2 as biomarkers.

Both proteins are involved in cell motility and proliferation, and in our opinion further studies are needed to verify the association between the expression of these proteins and tumor metastatization. In addition, PGAM2 being a glycolytic enzyme can be very useful in monitoring glycolysis as a fundamental metabolic pathway in cancer development.

In this study we demonstrate that uterine aspirates are a valuable biological fluid for the identification of cancer biomarkers.

The use of diagnostic biomarkers for EC can fasten the diagnostic process and reduce the sanitary costs.

Uterine aspirate may be an alternative to serum/plasma due to its high specificity [14].

In conclusion, our study expands the knowledge on the search for biomarkers in EC uterine aspirates. This study confirms the potential of 2-DE for biomarker discovery and western blotting for data verification. Proteins like ABRACL and PGAM2 have a great potential to reach the clinical phase after a validation phase.

## MATERIALS AND METHODS

### Patients, sample collection and treatment

A total of 16 patients (10 women suffering from EC and 6 non-EC controls) were recruited at the Institute for Maternal and Child Health – IRCCS “Burlo Garofolo” (Trieste, Italy) during 2014 and 2015. All procedures complied with the Declaration of Helsinki and were approved by the Technical and Scientific Committee of the Institute for Maternal and Child Health – IRCCS “Burlo Garofolo” (approval no. RC 19/08). All patients signed an informed consent. The clinical and pathological characteristics of the patients are described in Supplementary Table 1. Samples from control patients were obtained during the proliferative phase endometrium and the median age was 43 years. Samples from patients with endometrial cancer were obtained during postmenopause and the median age was 72.5 years.

Uterine aspirates were collected by aspiration with a Cornier Pipelle in the office of the clinician or in the operating room prior to surgery, and transferred to 10 ml microtubes and 3 ml of NaCl 0.9% was added with a protease inhibitor mixture (2 mM phenylmethylsulfonyl fluoride, 1 mM benzamidine, 1 mM EDTA and 10 mM NaF). The supernatant was centrifuged at 5,000 x g for 30min at 4°C followed by centrifugation at 15,000 x g for 30min at 4°C to remove cell debris. Approximately 3 ml of supernatant was desalted and concentrated using an Ultrafree-4 Centrifugal Filter Unit (EMD Millipore, Billerica, MA, USA) with a molecular weight cut-off of 3 kDa at 4,000 x g at 25°C until the remaining volume reached 50 μl. The protein content of the supernatant was determined by Bradford assay.

### 2-DE and image analysis

2-DE was performed like previously described [26]. For 2-DE analysis, 400 μg of proteins from each uterine aspirate sample was denatured in 300 μl of dissolution buffer [7 M urea, 2 M thiourea, 4% 3-[(3-cholamidopropyl)dimethylammonio]-1-propanesulfonate, 40 mM Tris, 65 mM dithiothreitol (DTT) and 0.24% Bio-Lyte 3/10 (Bio-Rad Laboratories, Inc., Hercules, CA, USA)] and a trace of bromophenol blue. ReadyStrip™ pH 3-10 Non-linear (NL) 18-cm immobilized pH gradient (IPG) strips (Bio-Rad Laboratories, Inc.) were rehydrated in a dissolution buffer at 50 V for 12h at 20°C, and isoelectric focusing (IEF) was performed in a PROTEAN IEF Cell (Bio-Rad Laboratories, Inc.). After the IEF, serial incubations were performed: first, the IPG strips were equilibrated for 20min in an equilibration buffer [6 M urea, 2% SDS, 50 mM Tris-HCl (pH 8.8), 30% glycerol and 1% DTT] and then in another equilibration buffer containing 4% iodoacetamide instead of DTT. For the second dimension, the equilibrated IPG strips were transferred to a 12% polyacrylamide gel. After electrophoresis, gels were fixed in 40% methanol and 10% acetic acid for 1h, and then stained for 16h with SYPRO Ruby. These gels, after SYPRO Ruby destaining, were stained for 48h with colloidal Coomassie Brilliant Blue. Double experimental replicates were performed per sample. Molecular weights were determined by comparison with Precision Plus Protein™ Prestained Standards (Bio-Rad Laboratories, Inc.), covering a range from 10 to 250 kDa. 2-DE gels were scanned with a Molecular Imager PharosFX System and analyzed using the Proteomweaver 4.0 software (both from Bio-Rad Laboratories, Inc.).

### Quantification of spot levels

2-DE image analysis was performed using the Proteomweaver 4.0 software. The analysis was performed by matching all gels from each uterine aspirate sample with a reference gel for the same condition, having the best resolution and the greatest number of spots, named “Master gel”.

Differences were considered to be significant when the ratio of the mean percentage of relative volume (%V), determined as %V = Vsingle spot/Vtotal spot, was ≥2.0 for up-regulated or ≤ 0.5 for down-regulated proteins, and the non-parametric Wilcoxon signed-rank test for matched samples resulted as being significant (P<0.05). Fold change was calculated as the ratio between the mean %V of the EC uterine aspirate and that of the normal uterine aspirate.

### Western blotting

Protein extracts (30 μg) used for 2-DE were separated by 12% or 15% SDS-PAGE and then transferred to a nitrocellulose membrane. The residual binding sites on the membrane were blocked by treatment with 5% dry milk in TBS-tween 20. After milk saturation the membrane was incubated overnight at 4°C with 1:200 diluted primary rabbit polyclonal antibody against ABRACL, 1:200 diluted primary rabbit polyclonal antibody against FGB, 1:1000 diluted primary rabbit polyclonal antibody against ANXA3, 1:300 diluted primary rabbit polyclonal antibody against PGAM2. The membrane was washed three times in TBST for 10min, and then incubated for 90min at 4°C with a horseradish peroxidase-conjugated anti-rabbit immunoglobulin G antibody (Sigma-Aldrich; Merck KGaA) at 1:3.000 dilution. Protein expression was visualized by chemiluminescence (SuperSignal West Pico Chemiluminescent Substrate; Thermo Fisher Scientific, Inc.), and the intensity of the signals was quantified by VersaDoc Imaging System (Bio-Rad Laboratories, Inc.). The intensities of the immunostained bands were normalized with the protein intensities measured by Coomassie blue (Sigma-Aldrich; Merck KGaA) from the same blot.

### Trypsin digestion and MS analysis

Spots from 2-DE were digested with sequencing grade-modified trypsin (Promega, Madison, WI, USA) and analyzed by mass spectrometry, as described by Ura *et al.* [27].

Spots extracted from 2-DE gels were washed with 50 mM NH_4_HCO_3_ and acetonitrile (ACN; Sigma-Aldrich) and dried under vacuum in a SpeedVac system. To each spot, three microliters of 12.5 ng/μl sequencing grade modified trypsin (Promega, Madison, WI, USA) in 50 mM NH_4_HCO_3_ were added, samples were allowed the rehydrate at 4°C and then covered with 50 mM NH_4_HCO_3_ and digested overnight at 37°C. Peptides were extracted with three changes of 50% ACN/0.1% formic acid (FA; Fluka), dried under vacuum and dissolved in 10 μl of 0.1% trifluoroacetic acid (TFA; Riedel-de Haën).

One microliter of matrix solution (α-cyano-4hydroxycinnamic acid 5 mg/ml in 70% acetonitrile/0.1% TFA) was mixed with 1 μl of each sample, and 0.8 μl of the final sample/matrix mixture was spotted onto a stainless steel MALDI target plate. Tandem mass spectrometry (MS/MS) analysis was performed on a MALDI-TOF/TOF 4800 mass spectrometer (AB Sciex, Framingham, MA, USA) in a data dependent mode: a full MS scan was acquired, followed by MS/MS spectra of the 10 most intense signals.

Samples that could not be identified by MALDI-TOF/TOF analysis were further analyzed by LC-MS/MS on an LTQ-Orbitrap XL mass spectrometer (Thermo Fisher Scientific, Rockford, IL, USA), coupled with a nano-HPLC Ultimate 3000 (Dionex – Thermo Fisher Scientific). The analysis was performed in a data dependent mode, and a full scan at 60,000 resolution on the Orbitrap was followed by MS/MS fragmentation scans on the 10 most intense ions acquired with CID fragmentation in the linear trap.

Data were converted into MGF (Mascot generic format) files to be elaborated with Proteome Discoverer 1.4 (Thermo Fisher Scientific), while raw data files from the LTQ-Orbitrap XL mass spectrometer were directly analyzed with the software. Proteome Discoverer was interfaced to a Mascot search engine, version 2.2.4 (Matrix Science, London, UK).

Enzyme specificity was set to trypsin with 1 missed cleavage. The database used for protein identification was UniProt Human (version 20150401; 90,411 sequences).

The tolerances were 50 ppm (parent) and 0.3 Da (fragment ions) for the MALDI-TOF/TOF data, and were set to 10 ppm for parent mass and to 0.6 Da for fragment ions for the files from LTQ-Orbitrap XL. Carbamidomethylation of cysteine residues was set to ‘fixed modification’ and methionine oxidation to ‘variable modification’.

A false discovery rate (FDR) was calculated by Proteome Discoverer based on the parallel search against a randomized database. Proteins were considered as positive hits if at least two independent peptides were identified with medium (95%) or high (99%) confidence.

### Functional analysis

The different abundant proteins identified were analyzed by PANTHER 11.0 (Protein Analysis through Evolutionary Relationships; http://www.pantherdb.org). Proteins were then classified according to their involvement in biological processes and molecular function. As most of the proteins take part in multiple processes, only the most relevant were reported.

### Statistical analysis

Statistical analyses were carried out with the non-parametric Wilcoxon signed-rank test for matched samples for both 2-DE and western blot data. P<0.05 was considered to indicate a statistically significant difference. All analyses were conducted with Stata/IC 14.1 for Windows (StataCorp LP, College Station, TX, USA).

## SUPPLEMENTARY MATERIALS TABLES



## Abbreviations

MALDI-TOF/TOF: Matrix-assisted laser desorption/ionization
LC-PRM: Targeted Proteomics by Parallel-Reaction Monitoring
LTQ Orbitrap XL: Hybrid Ion Trap-Orbitrap Mass Spectrometer
NaCl: Sodium chloride
EDTA: Ethylenediaminetetraacetic acid
NaF: Sodium fluoride
SDS: Sodium Dodecyl Sulphate
Tris-HCL: 2-Amino-2-(hydroxymethyl)-1,3-propanediol hydrochloride
IPG: Immobilized pH gradient
SDS-PAGE: Sodium Dodecyl Sulphate - Poly-Acrylamide Gel Electrophoresis
NH_4_HCO_3_: Ammonium bicarbonate
LC-MS/MS: Liquid chromatography–mass spectrometry
