# Supplementary material for: A proteomic approach for the identification of biomarkers in endometrial cancer uterine aspirate
Source: Oncotarget. 2017 Nov 30;8(65):109536–45. doi: 10.18632/oncotarget.22725 (PMC5752540; doi:10.18632/oncotarget.22725)
Supplement: Supplementary file 1 [file oncotarget-08-109536-s001.pdf]

A proteomic approach for the identification of biomarkers in endometrial cancer uterine aspirate

SUPPLEMENTARY MATERIALS

Supplementary Table 1: Clinico-pathological characteristics of the 16 women enrolled in the study

Sample type	Age	Diagnosis	Grade
Control	44	Hyperplastic endometrium	
Control	36	Normal Endometrium	
Control	42	Normal Endometrium	
Control	44	Endometrial polyp	
Control	42	Normal Endometrium	
Control	44	Normal Endometrium	
Tumor	69	Endometrioid adenocarcinoma	G2
Tumor	60	Endometrioid adenocarcinoma	G1
Tumor	86	Endometrioid adenocarcinoma	G3
Tumor	68	Endometrioid adenocarcinoma	G2
Tumor	71	Endometrioid adenocarcinoma	G2
Tumor	77	Endometrioid adenocarcinoma	G2
Tumor	67	Endometrioid adenocarcinoma	G2-G3
Tumor	77	Endometrioid adenocarcinoma	G2
Tumor	75	Endometrioid adenocarcinoma	G2
Tumor	74	Serous papillary adenocarcinoma	

Supplementary Table 2: For single spots, cut-offs for sensitivity and specificity for the identification of the diseased

SPOT	Cut-off	Sensitivity	Specificity	AUC	95%CI	p-value*
1	<0.20<br><1.00	75.0%<br>87.5%	100%<br>75.0%	0.898	0.732 - 1.000	0.007<br>0.041
2	<0.35	87.5%	75.0%	0.836	0.610 – 1.000	0.041
3	≥0.13	100%	100%	1.000	1.000 – 1.000	0.002
4	≥0.02<br>≥0.08	100%<br>83.3%	66.7%<br>100.%	0.944	0.816 – 1.000	0.061<br>0.015
5	≥0.04	100%	87.5%	0.953	0.851 – 1.000	0.000
6	≥0.13	100%	100%	1.000	1.000 – 1.000	0.008
7	≥0.02	100%	100%	1.000	1.000 – 1.000	0.008
8	≥0.10	100%	100%	1.000	1.000 – 1.000	0.002
9	≥0.11	100%	100%	1.000	1.000 – 1.000	0.008
10	≥0.20	100%	100%	1.000	1.000 – 1.000	0.002
11	≥0.12	100%	70.0%	0.910	0.787 – 1.000	0.003
12	≥0.09	100%	100%	1.000	1.000 – 1.000	0.008
13	≥0.0015	100%	100%	1.000	1.000 – 1.000	0.008
14	≥0.03	100%	100%	1.000	1.000 – 1.000	0.008
15	≥0.09	100%	100%	1.000	1.000 – 1.000	0.008
16	≥0.03<br>≥0.15	100%<br>71.4%	42.9%<br>71.4%	0.755	0.486 – 1.000	0.192<br>0.286
17	≥0.03<br>≥0.07	100%<br>83.3%	66.7%<br>83.3%	0.917	0.759 – 1.000	0.061<br>0.080
18	≥0.06	100%	100%	1.000	1.000 – 1.000	0.008
19	≥0.20	100%	100%	1.000	1.000 – 1.000	0.008
20	≥0.12	100%	100%	1.000	1.000 – 1.000	0.008
21	≥0.12	100%	100%	1.000	1.000 – 1.000	0.000
22	≥0.20<br>≥0.26<br>≥0.32	100%<br>77.8%<br>55.6%	33.3%<br>44.4%<br>88.9%	0.741	0.505 – 0.977	0.206<br>0.620<br>0.131
23	≥0.05	100%	100%	1.000	1.000 – 1.000	0.002
24	≥0.12<br>≥0.17	85.7%<br>71.4%	85.7%<br>100%	0.929	0.803 – 1.000	0.029<br>0.021
25	≥0.10	100%	100%	1.000	1.000 – 1.000	0.001
26	≥0.40<br>≥0.60	100%<br>88.9%	33.3%<br>55.6%	0.747	0.511 – 0.983	0.206<br>0.131

*p-value calculated with two-tailed Fisher exact test.
